# Bamboo Flowering from the Perspective of Comparative Genomics and Transcriptomics

**DOI:** 10.3389/fpls.2016.01900

**Published:** 2016-12-15

**Authors:** Prasun Biswas, Sukanya Chakraborty, Smritikana Dutta, Amita Pal, Malay Das

**Affiliations:** ^1^Plant Genomics Laboratory, Department of Life Sciences, Presidency UniversityKolkata, India; ^2^Division of Plant Biology, Bose InstituteKolkata, India

**Keywords:** bamboo, flowering pathways, genes, drought, plant age, future research

## Abstract

Bamboos are an important member of the subfamily Bambusoideae, family Poaceae. The plant group exhibits wide variation with respect to the timing (1–120 years) and nature (sporadic vs. gregarious) of flowering among species. Usually flowering in woody bamboos is synchronous across culms growing over a large area, known as gregarious flowering. In many monocarpic bamboos this is followed by mass death and seed setting. While in sporadic flowering an isolated wild clump may flower, set little or no seed and remain alive. Such wide variation in flowering time and extent means that the plant group serves as repositories for genes and expression patterns that are unique to bamboo. Due to the dearth of available genomic and transcriptomic resources, limited studies have been undertaken to identify the potential molecular players in bamboo flowering. The public release of the first bamboo genome sequence *Phyllostachys heterocycla*, availability of related genomes *Brachypodium distachyon* and *Oryza sativa* provide us the opportunity to study this long-standing biological problem in a comparative and functional genomics framework. We identified bamboo genes homologous to those of *Oryza* and *Brachypodium* that are involved in established pathways such as vernalization, photoperiod, autonomous, and hormonal regulation of flowering. Additionally, we investigated triggers like stress (drought), physiological maturity and micro RNAs that may play crucial roles in flowering. We also analyzed available transcriptome datasets of different bamboo species to identify genes and their involvement in bamboo flowering. Finally, we summarize potential research hurdles that need to be addressed in future research.

## Introduction

Flowering is one of the most important adaptations in the evolution of land plants. Numerous studies have been performed on annual, herbaceous model plants from dicotyledonous (*Arabidopsis, Antirrhinum*) and monocotyledonous (*Oryza*) groups to identify and characterize important floral pathway genes (Putterill et al., 2004; Colasanti and Coneva, 2009). However, the majority of commercially important plants are perennial and there remains a gap in translating knowledge gained from annual, model plants to perennial plants. Therefore, increasing research attention is being paid to perennial plants. While poplar (Jansson and Douglas, 2007) and white spurge have emerged as model perennial dicotyledonous plants (Anderson et al., 2007), research on perennialism remains elusive in monocots.

Bamboos are an important member of subfamily Bambusoideae, family Poaceae (Kellogg, 2015). Wide variations exist across bamboo species with respect to the flowering time, ranging from annual flowering to flowering after 120 years of vegetative growth (Janzen, 1976). There are even species for which the flowering time is not yet known. Variations in flowering time are not only diverse among species, but also at the population level. For instance, in the case of gregarious flowering all the individuals of a species growing over a wide geographical area bloom within a brief interval of time, and then all die after flowering (Nadgauda et al., 1997; Bhattacharya et al., 2009; Marchesini et al., 2009; Austin and Marchesini, 2012; Chaubey et al., 2013; Xie et al., 2016). In contrast, for sporadic flowering only a few culms of a population flower at a time (Ramanayake and Yakandawala, 1998; Bhattacharya et al., 2006; Xie et al., 2016). Such a wide variation in flowering time and extent indicates that the plant group serves as a repository for a wide range of genes and expression patterns that support such a life style. The ecological consequences of bamboo flowering, such as changes in dynamics of neighboring plant populations (Sertse et al., 2011), and impacts on endangered animals that depend on bamboo shoots (Reid et al., 1991; Azad-Thakur and Firake, 2014) have been topics of active research over decades. In comparison, the molecular aspects of bamboo flowering remain at a nascent stage. Studies have been conducted to characterize a limited number of flowering genes in different bamboo species such as *MADS18* from *Dendrocalamus latiflorus* (Bo et al., 2005), *FLOWERING LOCUS T (FT)* from *P. meyeri* (Hisamoto et al., 2008), *TERMINAL FLOWER 1 (TFL1)* like gene from *Bambusa oldhamii* (Zeng et al., 2015), *FRIGIDA (FRI)* from *P. violascens* (Liu et al., 2015), *MADS1* and *MADS2* from *P. praecox* (Lin et al., 2009), 10 genes related to floral transition and meristem identity in *D. latiflorus* (Wang et al., 2014) and 16 *MADS* box genes from *B. edulis* (Shih et al., 2014). Such targeted approaches are being complemented by high-throughput approaches, namely, *de novo* transcriptome sequencing and suppression subtractive hybridization (Lin et al., 2010; Liu et al., 2012; Zhang et al., 2012; Peng et al., 2013; Gao et al., 2014; Ge et al., 2016; Wysocki et al., 2016; Zhao et al., 2016).

The main aim of this article is to consider the current status of molecular understanding of bamboo flowering from the perspective of comparative genomics and transcriptomics. We queried the only sequenced genome of a temperate bamboo, *P. heterocycla* syn. *P. edulis*, to identify marker genes in established floral pathways (e.g., photoperiodic, vernalization, hormonal, and autonomous) and the influence of additional factors such as drought stress and physiological maturity. *P. edulis* is a diploid, temperate bamboo with chromosome number 2n = 48 and having a genome size of 2.075 Gb (Gui et al., 2007; Peng et al., 2013). In addition, we also explored transcriptome datasets of available bamboo taxa to assess their possible role in bamboo flowering. Finally, we have identified challenges that need to be overcome to understand what triggers bamboo flowering, the genetic controls of flowering, and the effects of gregarious monocarpic flowering cycles on bamboo evolution.

## Bamboo genes related to established flowring pathways

Depending on the nature of environmental or endogenous cues, flowering pathways can be broadly classified into vernalization (cold responsive), photoperiodic (day length responsive), autonomous (endogenous factors) and hormonal pathways.

## Vernalization pathway

In the model monocot *Oryza* the important vernalization genes are *VERNALIZATION 1* (*VRN1*), *VERNALIZATION INSENSITIVE LIKE 2*, and *3* (*VIL 2, 3*). An additional vernalization sensitive gene *VRN2* was isolated from *Triticum* (Dubcovsky et al., 2006), while its *Brachypodium* homolog *BdVRN2L* is vernalization insensitive (Ream et al., 2014). BLAST analyses have identified multiple copies of *OsVRN1, OsVIL2*, and *OsVIL3* homologs in *P*. *heterocycla* genome, but the homolog of *VRN2* remained undetected (Table 1). In order to understand their possible involvement in bamboo flowering, all available floral transcriptomes were searched. *VRN1* was detected in the shoot tissue specific EST library of *B. oldhamii* (Lin et al., 2010), while *VIN3* was identified from the floral transcriptomes of *P. heterocycla* (Peng et al., 2013) and *D. latiflorus* (Zhang et al., 2012). Another important vernalization gene, *At.FLC*, performs cold-mediated suppression of the floral activator *At.FT* during the seasonal transition from fall to winter (Michaels and Amasino, 1999). However, during prolonged cold exposure in winter, *FLC* activity is gradually down-regulated by *VRN1, VRN2*, and *VIN3* so that flowering is delayed until spring (Levy et al., 2002; Sung and Amasino, 2004). It was believed that *FLC*-like genes are absent in monocot plants (Choi et al., 2011), but recently two major *FLC* clades, namely, *MADS37* and *MADS51* genes, were identified in the temperate grass *Brachypodium distachyon* (Ruelens et al., 2013). Our BLAST analyses, however, could not detect *MADS37* or *MADS51* homologs in *P. heterocycla* at the set criterion of e^−40^, identity ≥50% and length coverage ≥60% of the query sequence (Table 1).

**Table 1:** **Identification of important flowering gene homologs in the model temperate grass- *Brachypodium distachyon* and temperate bamboo- *Phyllostachys heterocycla* using *Oryza sativa* amino acid sequences as query in BLAST-P analyses**.

**Flowering pathways/regulator**	**Genes**	***O. sativa* identifiers used as query**	**BLAST hits in *B. distachyon***	**BLAST hits in *P. heterocycla***
Vernalization	*VRN1*	*Os03g54160*	*Bradi1g08340* *Bradi1g59250*	*PH01000606G0250* *PH01000222G1190*
	*VIL2*	*Os12g34850*	*Bradi4g05950* *Bradi2g36237*	*PH01000006G3670*
				*PH01000674G0720*
				*PH01000258G0590* *PH01001556G0190*
	*VIL3*	*Os02g05840*	*Bradi3g04140*	*PH01000836G0140*
			*Bradi1g33450*	*PH01000114G1300* *PH01002795G0050*
	*FLC/MADS37*	n.f.c	*Bradi3g41297*	No hit
	*FLC/MADS51*	*Os01g69850*	*Bradi2g59191*	No hit
		n.f.c	*Bradi2g59119*	No hit
Photoperiod	*PHY A*	*Os03g51030*	*Bradi1g10520* *Bradi1g10510* *Bradi1g08400*	*PH01000222G1330* *PH01000606G0390*
	*PHY B*	*Os03g19590*	*Bradi1g64360* *Bradi1g08400*	*PH01000013G2240* *PH01000013G2230* *PH01000222G1330* *PH01000606G0390*
	*CRY 1*	*Os02g36380*	*Bradi3g46590* *Bradi5g11990* *Bradi3g49204*	*PH01000349G1020* *PH01000968G0540* *PH01002373G0140* *PH01000263G1210* *PH01002304G0120*
	*CRY2*	*Os02g41550*	*Bradi3g49204* *Bradi5g11990* *Bradi3g46590*	*PH01000968G0540* *PH01000349G1020* *PH01002304G0120* *PH01002373G0140* *PH01002304G0180*
	*CCA1*	*Os08g06110*	*Bradi3g16515*	*PH01001283G0510* *PH01000383G0300*
	*ELF 3*	*Os01g38530*	*Bradi2g14290*	*PH01000391G0450* *PH01000410G0960*
	*ELF 4*	*Os11g40610*	*Bradi4g13227* *Bradi1g60090*	*PH01002557G0050*
	*TOC 1*	*Os02g40510*	*Bradi3g48880*	*PH01003618G0130* *PH01000345G0790*
	*COP 1*	*Os02g53140*	*Bradi3g57667*	*PH01000928G0310* *PH01000311G0870*
	*FKF 1*	*Os11g34460*	*Bradi4g16630* *Bradi1g33610* *Bradi3g04040*	*PH01002958G0010* *PH01000114G1110* *PH01000836G0340* *PH01002213G0250* *PH01007024G0030*
	*ZTL*	*Os06g47890*	*Bradi1g33610* *Bradi3g04040* *Bradi4g16630*	*PH01007024G0030* *PH01002213G0250* *PH01000836G0340* *PH01000114G1110* *PH01002958G0010*
	*CO*	*Os06g16370*	*Bradi1g43670* *Bradi3g56260*	*PH01005551G0030*
	*GI*	*Os01g08700*	*Bradi2g05226*	*PH01002142G0290* *PH01001722G0270*
Autonomous	*FCA*	*Os09g03610*	*Bradi4g08727*	*PH01002230G0270*
	*FY*	*Os01g72220*	*Bradi2g60817*	*PH01001355G0380* *PH01002367G0110* *PH01002367G0090*
	*FLD*	*Os04g0560300*	*Bradi5g18210* *Bradi3g58720*	*PH01000272G0440*
	*FPA*	*Os09g0516300*	*Bradi4g35250*	*PH01000191G0930*
	*FVE*	*Os01g0710000*	*Bradi2g47940*	*PH01000048G0850* *PH01000241G0710*
	*LD*	*Os01g70810*	*Bradi2g59937*	*PH01006816G0010*
	*FLK*	*Os12g40560*	*Bradi4g02690* *Bradi1g14320*	*PH01000025G1210*
Gibberellic acid	*GA1*	*Os02g17780*	*Bradi2g33686*	*PH01000557G0660* *PH01002827G0080* *PH01004049G0170*
	*KAO*	*Os06g02019*	*Bradi1g51780* *Bradi1g30807* *Bradi5g00467* *Bradi4g05240*	*PH01000083G0900* *PH01003454G0070* *PH01000246G0620*
	*GA2ox1*	*Os05g06670*	*Bradi2g34837* *Bradi2g12440*	*PH01000685G0370*
	*GA2ox2*	*Os01g22910*	*Bradi2g12440* *Bradi2g34837*	*PH01000685G0370*
	*GA2ox3*	*Os01g55240*	*Bradi2g50280* *Bradi2g19900* *Bradi2g16750* *Bradi2g16727* *Bradi2g32577* *Bradi2g06670*	*PH01000018G1890* *PH01001124G0470* *PH01001567G0040* *PH01000273G0650* *PH01000274G0980*
	*GA3ox1*	*Os05g08540*	*Bradi2g04840* *Bradi4g23570*	*PH01002274G0400*
	*GA3ox2*	*Os01g08220*	*Bradi2g04840* *Bradi4g23570*	*PH01002274G0400*
	*GID1*	*Os05g33730*	*Bradi2g25600*	*PH01001316G0350* *PH01002734G0310*
	*GID2*	*Os02g36974*	*Bradi3g46950*	No hit
	*GAMYB*	*Os01g59660*	*Bradi2g53010*	*PH01000009G0060* *PH01000029G1950*
Integrator	*FT*	*Os06g06320/Hd3a*	*Bradi1g48830* *Bradi2g07070* *Bradi5g14010* *Bradi3g48036* *Bradi2g49795* *Bradi1g38150* *Bradi2g19670* *Bradi4g39730* *Bradi4g39760* *Bradi3g08890* *Bradi4g39750* *Bradi4g42400* *Bradi3g44860* *Bradi5g09270* *Bradi1g42510*	*PH01002288G0050* *PH01001134G0390* *PH01003363G0220* *PH01002570G0010*
		*Os06g06300/RFT1*	*Bradi1g48830* *Bradi2g07070* *Bradi3g48036* *Bradi5g14010* *Bradi2g49795* *Bradi2g19670* *Bradi3g08890* *Bradi1g38150* *Bradi4g39730* *Bradi4g39760* *Bradi4g42400* *Bradi4g39750* *Bradi4g35040* *Bradi3g44860* *Bradi5g09270* *Bradi2g27860* *Bradi2g01020*	*PH01002288G0050* *PH01001134G0390* *PH01003363G0220* *PH01002570G0010* *PH01007086G0020*
	*SOC1 /MADS50*	*Os03g03070*	*Bradi3g32090* *Bradi1g77020* *Bradi3g51800*	*PH01000759G0450* *PH01000059G1270* *PH01000107G0570* *PH01002152G0120*
Drought	*Dof12*	*Os03g07360*	*Bradi1g73710* *Bradi3g25670*	*PH01000113G0300* *PH01000188G0230* *PH01000219G0080* *PH01001264G0440*
Physiological maturity	*LFY*	*Os04g51000*	*Bradi5g20340*	No hit
	*TFL1*	*Os11g05470/RCN1*	*Bradi4g42400* *Bradi5g09270* *Bradi3g44860 Bradi1g48830* *Bradi2g07070 Bradi3g48036* *Bradi2g49795 Bradi5g14010* *Bradi2g19670* *Bradi3g08890* *Bradi2g01020* *Bradi1g38150 Bradi4g39730*	*PH01001134G0390* *PH01003363G0220* *PH01002570G0010* *PH01007086G0020* *PH01002288G0050*
		*Os12g05590/RCN3*	*Bradi4g42400* *Bradi5g09270* *Bradi3g44860* *Bradi1g48830* *Bradi2g07070* *Bradi3g48036* *Bradi2g49795* *Bradi5g14010* *Bradi2g19670* *Bradi3g08890* *Bradi2g01020* *Bradi1g38150 Bradi4g39730*	*PH01001134G0390* *PH01003363G0220* *PH01002570G0010* *PH01007086G0020* *PH01002288G0050*

*The criteria used were: e^−40^, identity = 50% and length coverage = 60% of the query sequence. If the O. sativa gene is yet to be functionally characterized (no functional characterization, n.f.c), B. distachyon gene sequences were used as query. When no homologous sequences were identified in our set criteria, it is mentioned as no hit*.

## Photoperiodic pathway

In the photoperiodic pathway, the circadian rhythm of light and dark periods plays a major role in flower initiation. In *Oryza* a series of genes that include *PHYTOCHROMES A* and *B (PHYA* and *PHYB), CRYPTOCHROMES 1* and *2 (CRY1* and *CRY2*), *CIRCADIAN CLOCK ASSOCIATED 1 (CCA1), EARLY FLOWERING 4 (ELF4), TIMING OF CAB EXPRESSION 1 (TOC1), CONSTITUTIVE PHOTOMORPHOGENIC 1 (COP1), EARLY FLOWERING 3 (ELF3), GIGANTEA (GI), FLAVIN-BINDING KELCH REPEAT F BOX 1(FKF1)* and *ZEITLUPE* (*ZTL*) receive the circadian signal and transfer it to *CONSTANS* (*CO*) for further downstream regulation. Our BLAST analyses identified at least one one homologous copy of each of these genes in the queried *P. heterocycla* genome (Table 1). ESTs homologous to *CRY1, CRY2, PHY, FKF1, COP1, ELF3, ELF4, GI, CCA1*, and *CO* were found in the floral transcriptomes of *P. edulis, B. oldhamii*, and *D. latiflorus*, suggesting their role in bamboo flower induction (Lin et al., 2010; Zhang et al., 2012; Peng et al., 2013; Gao et al., 2014). The transcriptional expression level of *CO* varied across libraries. For instance, it was low in *P. edulis* and correlated with the presence of *L1* and *GYPSY* transposable elements in the regulatory region of the gene (Peng et al., 2013). On the other hand, a high level of *CO* expression was obtained in the floral tissues of *D. latiflorus* (Zhang et al., 2012). *CO*, along with the *CCAAT* box binding factor (*NFY*), bind to the *CCAAT* box of *FT* promoter and result in flowering (Ben-Naim et al., 2006). Therefore, the co-expression of *CO* and *FT* (i.e., *CO-FT* regulon) plays a crucial role in the regulation of flowering time. Our BLAST analyses identified 5 *FT-like* and 1 *CO-like* homologs in *P. heterocycla* (Table 1). Similarly, single or multiple *FT* copies have been identified and characterized in *D. latiflorus, P. meyeri*, and *P. violascens* (Hisamoto and Kobayashi, 2007, 2013; Hisamoto et al., 2008; Wang et al., 2014; Guo et al., 2015). Detailed expression analysis of *PmFT* revealed that its expression is primarily restricted to leaves, but highest during full bloom (Hisamoto and Kobayashi, 2013). Expression of the two *FT* genes and their functional diversification was reported in *P. violascens* (Guo et al., 2015). *PvFT1* is expressed in leaves and induces flowering, while *PvFT2* possibly plays a role in floral organogenesis. Another important floral integrator, *SUPRESSOR OF OVEREXPRESSION OF CONSTANS 1 (SOC1)*, was identified by our BLAST analyses (Table 1) and was also expressed in the floral transcriptomes of *P. edulis, Guadua inermis, Otatea acuminate, Lithachne pauciflora*, and *P. aurea* (Peng et al., 2013; Wysocki et al., 2016).

## Autonomous and hormonal pathway

In addition to environmental cues, additional flower inducing factors are present within a plant itself and are called endogenous or autonomous signals. This pathway is well studied in *Arabidopsis*, but is less characterized in monocot plants (Lee et al., 2005; Abou-Elwafa et al., 2011). The important genes are *FLOWERING LOCUS CA (FCA), FLOWERING LOCUS D (FLD), FLOWERING LOCUS KH DOMAIN (FLK), FLOWERING LOCUS PA (FPA), FLOWERING LOCUS VE (FVE), FLOWERING LOCUS Y (FY)*, and *LUMINIDEPENDENS* (LD, Simpson, 2004). These genes promote flowering by suppressing *FLC* expression (Simpson, 2004; Quesada et al., 2005). Our BLAST analyses identified one or more *P. heterocycla* homologs for the majority of these genes (Table 1), which were reported in the floral transcriptomes of *B. oldhamii* (Lin et al., 2010), *D. latiflorus* (Zhang et al., 2012), and *P. heterocycla* (Peng et al., 2013) and suggest possible roles in bamboo flowering.

The role of gibberellic acid (GA) in the induction of flowering is well established in *Oryza* (Kwon and Paek, 2016). Many important genes related to GA biosynthesis (*ent-KAURENE SYNTHETASE A- GA1, ent-KAURENOIC ACID OXIDASE-KAO, GA 2-OXIDASE*-*GA2ox, GA3ox*) and receptors (*GIBBERELLIN INSENSITIVE DWARF1- GID1, GID2)* have been characterized (Sakamoto et al., 2004). *GID1* and *GID2* are responsible for proteasome mediated *DELLA* degradation and promote flowering through upregulation of *GAMYB* (Kwon and Paek, 2016). At least one *P. heterocycla* homolog has been detected for the majority of these genes in our BLAST analyses (Table 1). The possible involvement of GA in bamboo flowering is supported by the identification of *GA1, SLY, GID1, GID2, GAMYB* ESTs in the floral transcriptome of *P. heterocycla* (Gao et al., 2014) and *D. latiflorus* (Zhang et al., 2012).

## Possible physiological and genetic factors regulating bamboo flowering

### Stress

Increasing evidence suggests a link between stress and bamboo flowering (Rai and Dey, 2012; Peng et al., 2013; Ge et al., 2016). Overall expression level of general stress responsive genes involved in ABA, ethylene, sugar metabolism and Ca^+2^ dependent signaling pathway were 11.1-fold higher than that of the flowering genes in *P. heterocycla* (Peng et al., 2013). Particularly, a few members of the DNA binding with one finger (*Dof*) transcription factor family were highly up-regulated in the floral transcriptome (Imaizumi et al., 2005). For instance, *Ph.Dof12* was about 16-fold up-regulated in the flowering tissues of *P. heterocycla* collected from a drought affected area (Peng et al., 2013). Similarly, 28 unigenes related to *Dof3, Dof4, Dof5, Dof12*, and *Cycling Dof Factors* (*CDF*) were detected in the floral transcriptome of *P. edulis* (Gao et al., 2014). The *Dof* family is composed of 15 genes in *Phyllostachys* and a comprehensive functional characterization of these genes may provide new insights. Particularly, analyzing the enrichment of the drought-responsive cis-elements in their promoter regions could identify candidate genes that are induced under drought conditions.

### Physiological maturity and micro RNAs

Scientific evidence emerging from research on various perennial plants suggests an important role of *TERMINAL FLOWER 1* (*TFL1)* and microRNAs (*miRNAs*) in maintaining a long vegetative phase (Huijser and Schmid, 2011). Our BLAST analyses identified five copies of *Ph.TFL1* genes in *P. heterocycla* (Table 1). A functional *TFL1* gene was isolated from *B. oldhamii* and was overexpressed in *Arabidopsis* (Zeng et al., 2015). The overexpressed lines showed delayed flowering, suggesting that *TFL1* may have a role in maintaining vegetative growth. In addition, *TFL1* may have an important function in differentiation of bamboo floral organs, as indicated by higher expression of *TFL1* in late floral developmental stages relative to early stages in *B. oldhamii* and *D. latiflorus* (Wang et al., 2014).

Long maintenance of the vegetative phase in the majority of bamboos can also be regulated at the post-transcriptional level, such as by miRNAs. In rice *miR156* is known to repress flowering by targeting *SQUAMOSA PROMOTER BINDING PROTEIN-LIKE* (*SBP/SPL*) transcription factor (*SPL*s, Xiong et al., 2006). Expression of *miR156* showed significant down-regulation through the transition from vegetative to flowering stages in *P. edulis* (Gao et al., 2015). Additional candidates that may have roles are *miR164a, miR166a, miR167a, miR535a, miR159a.1, miR164a*, and *miR168-3-p* (Gao et al., 2015; Ge et al., 2016). In contrast, some micro RNAs may play positive roles in bamboo flowering. One such candidate is *miR172*, which controls flowering time and the formation of floral organs through the regulation of the *AP2*-like transcription factor (Lee et al., 2014). *miR172a* showed an increase in expression level during progression from vegetative to the flowering phase in *P. edulis* (Gao et al., 2015). The expression of other miRNAs such as *miR169b, miR395h-5p*, and *miR529-3p* were higher in floral tissues than in vegetative tissues.

## Future challenges

### Appropriate tissue sampling

**Figure 1:**
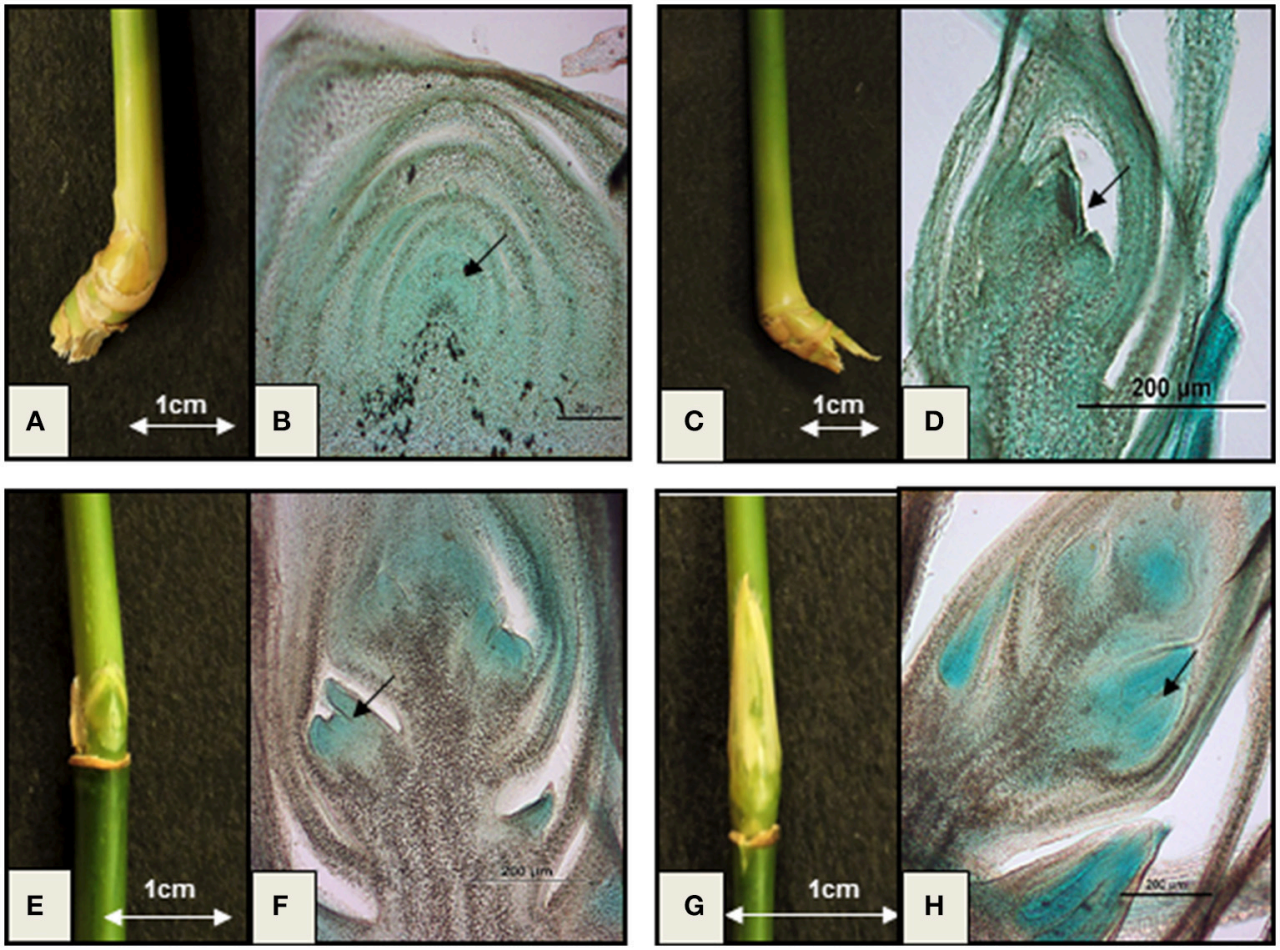
**Important vegetative and floral developmental stages *of B. tulda*. (A)** External morphology of nodal vegetative bud (~0.6 × 0.7 cm in dimension); **(B)** Longitudinal section (L.S.) of vegetative bud. The shoot apical meristem (SAM) is dome shaped (marked with arrow); **(C)** External morphology of an early stage inflorescence bud (~0.3 × 0.3 cm in dimension); **(D)** LS of the early stage inflorescence bud having triangular inflorescence meristem (marked with arrow); **(E)** External morphology of middle stage inflorescence bud (~0.8 × 0.5 cm in dimension); **(F)** LS of middle stage inflorescence bud showing differentiated floral primordia (marked with arrow); **(G)** External morphology of late stage inflorescence bud (~1.2 × 0.6 cm in dimension); **(H)** LS of late stage inflorescence bud having differentiated anther primordia (marked with arrow).

Identification of proper tissue stages is critical since the majority of flowering genes are transiently expressed soon before or after floral induction. Unlike *Arabidopsis* or *Oryza*, wild bamboo floral tissue stages are not easily traceable. Therefore, tissue culture methods have been tried to induce flowering and to study defined stages of induced floral transcriptomes of *B. oldhamii in vitro* (Lin et al., 2010). However, this study raised doubt about comparability of the transcription patterns under *in vitro* conditions vs. naturally occurring flowering. A large unigene set (146,395) generated from the floral transcriptomes of naturally grown *D. latiflorus* could not detect the important integrator gene *FT*, although it was detected in the transcriptome of *P. edulis*. This emphasizes the need to define *in vivo* floral stages with higher accuracy in order to make data generated by different research groups more comparable. Therefore, we studied the microscopic histology of different flowering stages of wild *B. tulda* plants and compared them with the external morphology of buds to identify phenotypic markers for specific growth stages (Figure 1). The external morphological features of nodal vegetative buds are indistinguishable from those of early stage inflorescence bud. However, this is one of the most crucial tissue stages with respect to the identification of genes involved in flower induction. Close observation of the early inflorescence bud revealed that it is slightly smaller in size, pale yellow in color, and bulged in the middle (Figures 1A,C). Histological analyses reveal that the shoot apical meristem of the nodal vegetative bud is dome shaped and covered with compactly arranged leaf primordia (Figure 1B). But the early staged inflorescence meristem is slightly smaller in size and triangular in shape (Figure 1D). The middle stage floral bud could be differentiated from the early stage by its elongated shape and bright green color (Figure 1E). Histological analysis revealed that it is composed of one or two floral primordia at the base of the rachis and an undifferentiated inflorescence meristem at the apex (Figure 1F). The late inflorescence bud is easily identifiable from all the other stages by its long and slender shape (Figure 1G). It is composed of three to four visible florets having differentiated anther primordia at the base of the rachis and an undifferentiated apical inflorescence meristem (Figure 1H).

### Gene family expansion, high sequence homology and associated challenges

Bamboos are highly polyploid plants with big genomes (2075 Mb for *P. heterocycla* compared to 125 Mb for *A. thaliana*). Consequently, the majority of genes are present in multiple copies. It would be important to dissect their evolutionary origin (orthologs-functional, paralogs-old/recent vs. tandem duplicates) and deduce their functional conservation or divergence by studying detailed transcriptional expression patterns (Das et al., 2016). However, the majority of these genes are very similar in sequence, which creates challenges in maintaining specificity in gene expression analyses. Example of this are *FT* and *TFL1* genes, which are members of the *Phosphatidylethanolamine-binding protein (PEBP)* family and share high sequence similarity (>60%). However, they are functionally antagonistic to each other. There are diagnostic amino acids, which are crucial to maintain either *FT* (Tyr-85) or *TFL1* (His-88) function (Hanzawa et al., 2005). Our BLAST analyses identified five *P. heterocycla* homologs each for *FT* and *TFL1* and they are completely overlapping with each other (Table 1). Follow-up analysis indicated *PH01002288G0050* as the predicted *FT* gene, while the other four, *PH01001134G0390, PH01003363G0220, PH01002570G0010, PH01007086G0020* are *TFL1*. Therefore, in addition to large-scale sequence analyses such as BLAST, individual gene sequences should be checked for correct gene function annotation.

### Genetic tools for functional validation

With the completion of gene sequencing and expression pattern characterization, the next challenge would be to confirm gene functions using loss- or gain-of-function mutants. This is especially important for multi copy genes for which expression data is not indicative of functional differentiation among copies. Therefore, a model plant is needed in which tissue culture and genetic transformation are easy to perform. Woody bamboos are generally recalcitrant and present several challenges (Das and Pal, 2005a). Since loss-of-function mutation analyses would be challenging, other model plants could be exploited to perform genetic complementation analyses by ectopically expressing bamboo flowering genes. Rice could be useful for such purposes due to its close evolutionary relationship, related floral biology and availability of mutant lines for several genes. However, many rice genes and associated mutant phenotypes have yet to be characterized.

### Development of a new model system for tropical bamboo

The majority of available research reports are on the tetraploid bamboo *Phyllostachys*, predominantly found in the temperate regions of China and Japan. However, enormous biodiversity is found in the tropical regions and dominated by members of the genus *Bambusa*. Therefore, the genome/transcriptomes of a tropical bamboo should be characterized. These have enormous economic importance, a large population size, wide genetic diversity (Das et al., 2008), molecular methods for species level identification (Das et al., 2005), a standardized micropropagation protocol (Das and Pal, 2005b), incidents of both gregarious (Mohan Ram and Harigopal, 1981) and sporadic flowering (Bhattacharya et al., 2006), which taken together makes *B. tulda* a good choice as a model species of tropical bamboos.

## Author contributions

MD and AP collaborated in this study. PB, SC, and SD had done the bioinformatics and histological analyses. MD wrote the paper with input from all co-authors.

### Conflict of interest statement

The authors declare that the research was conducted in the absence of any commercial or financial relationships that could be construed as a potential conflict of interest.
